# Telemedicine-Based Virtual Stone Clinics for Renal Colic: Cost-Benefit Insights and Adoption Barriers

**DOI:** 10.7759/cureus.97028

**Published:** 2025-11-17

**Authors:** Nazeer Ibraheem, Mohamed Mohamed, Momen Abdelglil, Muhammad Rakib Hasan, Mohammad Ekhlasur Rahman

**Affiliations:** 1 Urology, New Cross Hospital, The Royal Wolverhampton NHS Trust, Wolverhampton, GBR; 2 Radiology, Kettering General Hospital, Kettering, GBR; 3 Pediatric Surgery, Mansoura University Children's Hospital, Mansoura, EGY; 4 Urology, Watford General Hospital, West Hertfordshire Hospitals NHS Foundation Trust, Watford, GBR

**Keywords:** evaluation for telehealth, renal colic, stone clinics, telehealth, telemedicine (tm), urology, virtual urologic care

## Abstract

Telemedicine in urology has gained substantial attention, accelerating in adoption due to the COVID-19 pandemic and its potential to bridge significant gaps in healthcare access, particularly given that 62% of U.S. counties lack a urologist. This narrative review outlines its applications, cost benefits, and adoption barriers. Studies demonstrate high patient satisfaction, especially for postoperative consultations and prostate-specific antigen (PSA) tracking. Virtual care is highly effective for managing conditions like nephrolithiasis and benign ureteric colic; one quality improvement study focusing on ureteric colic successfully avoided 71.1% of face-to-face follow-ups while maintaining high safety and patient satisfaction (93.1%). The financial advantages are significant, with virtual stone clinics reducing waiting times and saving patients an average of $147 to $186 per visit by minimizing travel costs and time away from work. Despite these benefits, widespread adoption faces hurdles. Key challenges include a lack of patient trust in virtual sessions compared to in-person care, particularly among minority groups. Furthermore, technological barriers, such as inadequate digital literacy and a lack of broadband access, disproportionately affect elderly and ethnic minority populations, which risks exacerbating existing health disparities. Telemedicine is also limited by its unsuitability for conditions requiring a physical examination. Addressing these obstacles is essential to ensuring virtual care remains an affordable and equitable component of future healthcare.

## Introduction and background

Telemedicine is the delivery of healthcare when the patient and clinician are in different locations, using telecommunications (e.g., telephone or video) and digital transfer of clinical data, including imaging, to evaluate, manage, and follow up patients. This approach was first demonstrated in urology through real-time remote consultations with image review, and more recently through structured phone and video workflows that safely reduce in-person visits [1-5]. The use of telemedicine has been made possible by improvements in technology in many areas, especially with digital devices and communication [1,2]. When applied in surgical procedures, telemedicine has demonstrated different levels of success. Before the COVID epidemic, it was mostly used in surgery for cases where difficulties with accessibility were considered challenging [3,4]. Surgical practices rapidly adjusted during the pandemic, leading to an increase in telemedicine adoption [5,6]. Over the past 15 years, significant changes and technological advancements have been made in telehealth systems. Urology, along with other medical fields, has been a leader in successfully adopting this form of medical care [7,8].

Even before the pandemic, telemedicine was being used and highlighted in urology [2,9-11]. The American Urological Association (AUA) has even created an AUA Telemedicine Taskforce, composed of urologists who guide new telemedicine efforts within the organization [12]. Post-pandemic, data suggest high patient satisfaction with urology telemedicine appointments amongst older patients, for prostatectomy follow-up care and monitoring, and for prostate-specific antigen (PSA) surveillance [13]. Research in men's health and urology indicates the valuable role of telehealth and integrated care, especially as there is a rising incidence of conditions like benign prostatic hyperplasia, prostate cancer, and erectile dysfunction [11].

Telemedicine is adjusted and suitable for postoperative care and follow-up clinics, especially after minimally invasive surgeries [14]. Patients may conserve money, time, and travel for pre- and postoperative consultations, based on a systematic review of telemedicine in surgical care [15]. This narrative benefits clinicians, policymakers, and patients by summarizing evidence, highlighting workflows, clarifying gaps, and offering actionable recommendations, fostering equitable implementation and informed decision-making.

## Review

Urological care access

According to the AUA's 2023 census report, many countries do not have a urologist, and a quarter of these communities also lack good internet access for many people [16,17]. The increasing global population has not been matched by the same number of active urologists. It additionally results in a gap in access to healthcare for urological conditions, which can be covered by innovative approaches like telehealth, which provides remote consultations [18]. Urology providers expressed significant satisfaction with telemedicine during the COVID-19 pandemic, finding it convenient and efficient, and expressed a desire to continue using it with patients [19]

Integrating telehealth with urologic care can significantly address men's health apathy, urologist shortages, and access barriers, especially given that over 85 million American men worry about urologic health but often neglect preventive care [20]. There will likely be a severe scarcity of urologists to meet the increasing demands of an aging population, as 29.8% of the urology workforce is 65 years of age or older [21]. Access to urological treatment is enhanced by telehealth services. Patients are more likely to engage in this accessible treatment model because they benefit from flexible scheduling and easy, private, at-home testing [22].

Patients struggle to access urological care in many areas, as it is calculated that 61.4% of U.S. counties do not have a urologist, and fewer than 10% of primary urology practices are located outside major metropolitan areas [23]. The transmission of urological care has been totally altered by telemedicine, especially throughout rural regions where there are few qualified and experienced urologists [17,24,25].

**Figure 1:**
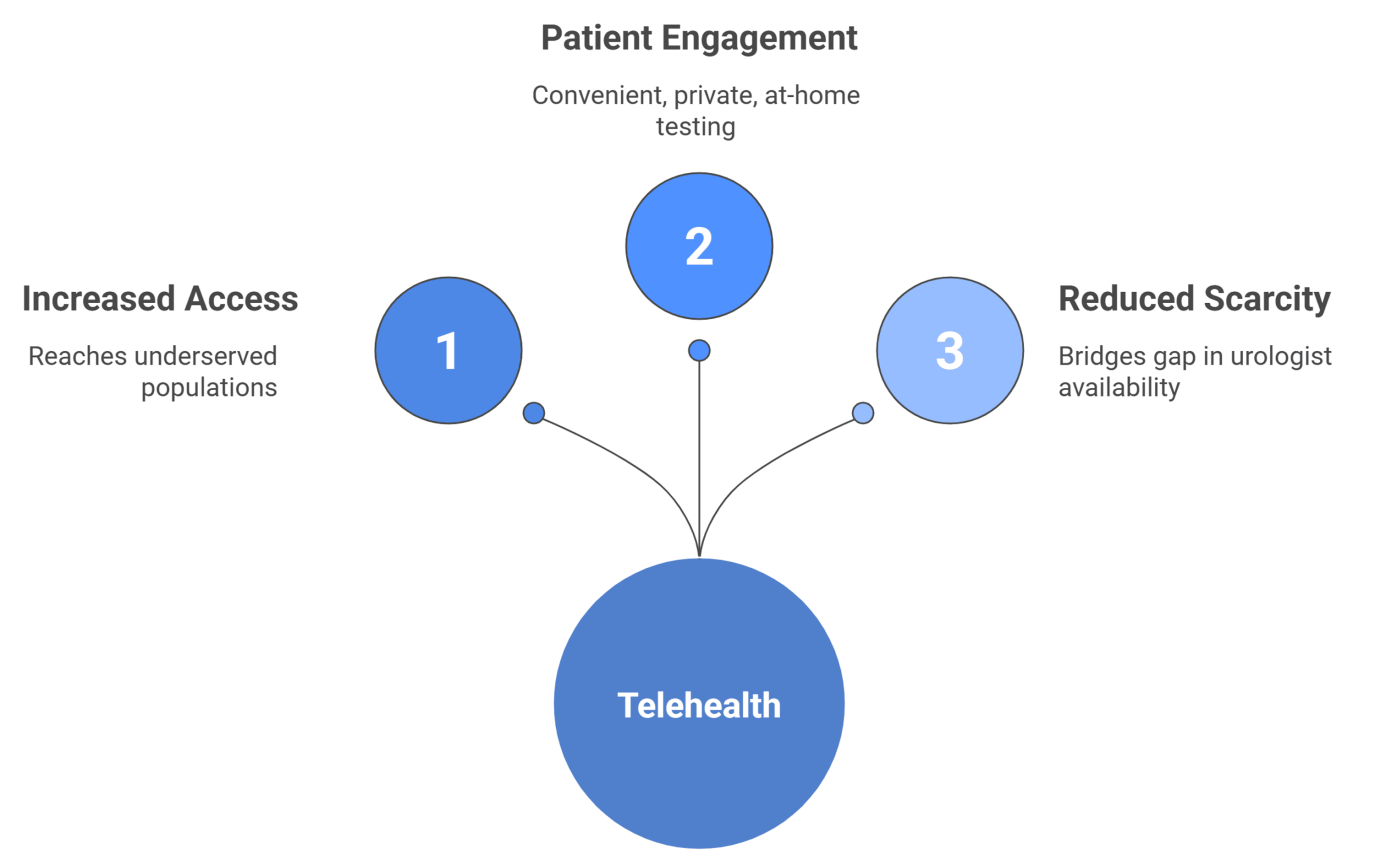
Urological care access. Image credit: Momen Abdelglil. Sources: [16-27].

A 2016 study discovered that veterans with hematuria regarded the initial telephone consultation or follow-up significantly (nine out of ten) for convenience, efficiency, and quality of care. The majority selected phone consultations over in-person meetings to reduce travel, and 97% of respondents gave a high-quality score, revealing that telemedicine is effective at removing barriers while preserving treatment standards (Figure 1) [26].

Current benefits of telemedicine in urology

Numerous studies indicate the beneficial effects of using video consultations in urology clinics for a variety of purposes, such as referrals for hematuria and follow-up appointments that follow for a wide range of conditions, like urinary tract infections, benign prostatic hyperplasia, and nephrolithiasis. This research indicates that many patients were satisfied and accepted of this telemedicine strategy [10,27,28]. 

Due to its generally benign nature, ureteric colic is one of the many urological disorders that doctors can monitor. Symptoms and scan results can be discussed remotely without direct examination, eliminating the need for an in-person follow-up. Telemedicine could also be beneficial for managing other urological issues, such as monitoring of renal cysts, kidney stones, and long-term management of lower urinary tract symptoms [7,29].

Ong et al. describe the implementation of a telemedicine service for ureteric colic patients to reduce unnecessary face-to-face consultations and shorten appointment waiting period to improve both patient and doctor experience. This quality improvement study, using the Plan-Do-Study-Act method, replaced in-person scan reviews with phone consultations for patients without high-risk features like fever or severe pain. Over three years, the service recruited 53.2% of eligible patients and successfully avoided 71.1% of face-to-face follow-up consultations, primarily because patients had normal scan results or did not attend their scan appointments. The mean time to review results was 30 days, with low six-month intervention (3.4%) and unplanned reattendance (3.2%) rates, indicating the service was safe and effective. Patient satisfaction was high at 93.1%, demonstrating that the telemedicine service sustainably reduced in-person visits without compromising patient safety [1]. 

A study by Zholudev et al. found that telemedicine offers several advantages over in-person consultations or face-to-face patient visits, including reduced direct costs and time, as well as indirect benefits such as more flexible scheduling, increased clinic and parking availability, less traffic, and lower greenhouse gas emissions [30,31]. Smith et al. and Connor et al. have documented the establishment of virtual stone clinics within the UK's National Health Service, demonstrating advantages such as reduced waiting times for initial appointments, financial savings, and fewer environmental problems [32,33].

Cost Saving

Initially, some healthcare authorities and professionals were concerned that telemedicine technologies would exacerbate health disparities, as telehealth companies seemed to prioritize well-resourced places and patients. However, simpler telemedicine methods like phone calls can effectively provide specialized care to individuals in low-income communities so that we can treat these financial problems [25,34]. 

Detecting prostate cancer at an early stage through telehealth consultations can result in significant cost savings. Compared to a later diagnosis (stage 4), some recorded upwards of $309,000 over four years [35]. When the cost is taken into consideration, this emphasizes the possible importance of telemedicine in an array of urological concerns.

Based on a review, telemedicine helped patients with certain urological diseases cost an average of $149 to $252 per visit. Videoconferencing was preferred by patients, indicating that more successful telehealth interactions result in these cost savings; also, patients accepted it [36]. A study was performed at a comprehensive cancer center designated by the National Cancer Institute. Due to lower travel costs and less time spent away from work, patients under 65 who completed about 25,500 telehealth assessments and care saved an average of $147 to $186 each visit [37]. 

Costs per patient in a clinic were successfully lowered by 69% using one technique. This approach improved patient family satisfaction and tripled the number of patients seen in a clinic day (from 14 to 43). These findings illustrate the important operational and economic benefits that integrated care can offer the medical field [38].

Checcucci et al. conducted a study in Italy during the COVID-19 pandemic, assessing the effectiveness of phone-call telemedicine for following up with 607 patients experiencing benign urological conditions after their in-person appointments were canceled to monitor their condition and suggest a follow-up process. He assessed the patients' health as well as how they felt about this telemedicine strategy. Important results showed that 87.5% of patients had stable symptoms and didn't need to see a doctor in person. Additionally, 81.5% of patients were more worried about the possibility of COVID-19 infection than they were about their urological disease. Although patients thought the phone visits were very helpful and easy to understand, a major drawback was that 53% of them lacked the technology required for more sophisticated televisits. The authors concluded that this telemedicine approach successfully lowers needless hospital visits [39].

Community perspectives

Several groups within every healthcare system are at a higher risk of receiving unfavorable health news. These groups may now experience additional difficulties due to insufficient digital literacy or limited access to these digital devices. This obstacle disproportionately affects rural communities, the elderly, ethnic/racial minority populations, individuals with low socioeconomic status, those with inadequate health education, and people with limited English proficiency [40-42]. 

Specialized virtual consultations are increasingly essential in rural and underserved areas experiencing significant workforce shortages that will help them to provide better care. They are crucial for improving patient access to necessary care and provide a more efficient and safer option than traditional in-person appointments [25,43]. 

Some health issues can lead to feelings of shame, isolation, and low self-worth, impacting one's health-related quality of life; they may also prevent the patient from seeking help [44-46]. Urgency urinary incontinence (UUI)and mixed urinary incontinence (MUI) significantly impacts many women's quality of life, as highlighted by literature that mobile apps are effective remote monitoring methods. A study by Wadensten et al. found that a mobile testing application was effective for self-monitoring in women with UUI and MUI, and most participants reported being satisfied with their treatment [47].

Shin et al. assessed patient satisfaction and cost savings from telemedicine visits in which they surveyed women who completed these visits and found that 89.8% of respondents were satisfied. Many cases saved at least an hour, and over a quarter saved more than $25 on transportation and other means. However, 52.9% of televisits still required a follow-up in person, particularly for chief complaints of prolapse, new patients, and Hispanic ethnicity [48].

Efthymiadis et al. assessed patient satisfaction with telephone consultations using a modified Telehealth Satisfaction Scale (TeSS) by testing patients over one month. Responses were examined according to gender, age, and clinic type. *Excellent *or *Agree* remarks were more frequently reported by patients from PSA surveillance clinics and post-radical prostatectomy clinics. Gender did not affect the outcomes; however, older patients were more likely to answer *agree* to a particular item. The study found that most urological patients were happy with phone consultations, emphasizing the advantages of remote consultations for both patients and physicians [13].

A previous study revealed that common sociodemographic characteristics, such as an individual's race or ethnicity and country of birth, showed no discernible correlation with their likelihood of attending a telehealth appointment or the success of these tools [49]. This indicates that the widespread adoption is not being significantly impeded by such specific demographics.

Another study found no differences in patient satisfaction across a wide range of demographics, including the old people. This implies that these already underprivileged groups may be disproportionately impacted by the difficulties presented by technology, which would increase already-existing gaps in healthcare access and quality [50]. Video appointments for telemedicine were found to be less frequently utilized by individuals of Black and Latinx ethnicities, exacerbating existing disparities [51].

Bell et al. reported that married or partnered patients attended 88 appointments (38.6%), a significantly higher proportion than the 18 appointments attended by single patients (19.2%) (*P* = 0.001). Factors associated with lower attendance in a multivariable analysis included being single or divorced, having an active substance use problem, and being a new patient. Additionally, using a language other than English and having Medicaid insurance were independently linked to fewer or noncontinuous telehealth visits [49].

A study by Kim et al. explored whether follow-up phone calls by non-medical professionals after pediatric urological surgery could improve patient satisfaction and reduce unnecessary healthcare use and visits, which made financial burdens, and they suggested that clear perioperative guidelines and protocols might be more impactful than postoperative phone calls in influencing patient and family satisfaction, and that such calls within 48 hours of surgery may not significantly affect families undergoing same-day pediatric urology surgery [52].

Gan et al.'s research found that families liked video consultations, with a satisfaction score of 10/10, suggesting these visits adequately addressed their children's needs and enhanced their satisfaction. A significant 90% of families endorsed telehealth visits. Technical issues were minimal, with only 15.6% of families reporting visual or hearing difficulties and 7.6% experiencing internet problems [53].

Challenges for the adoption

**Figure 2:**
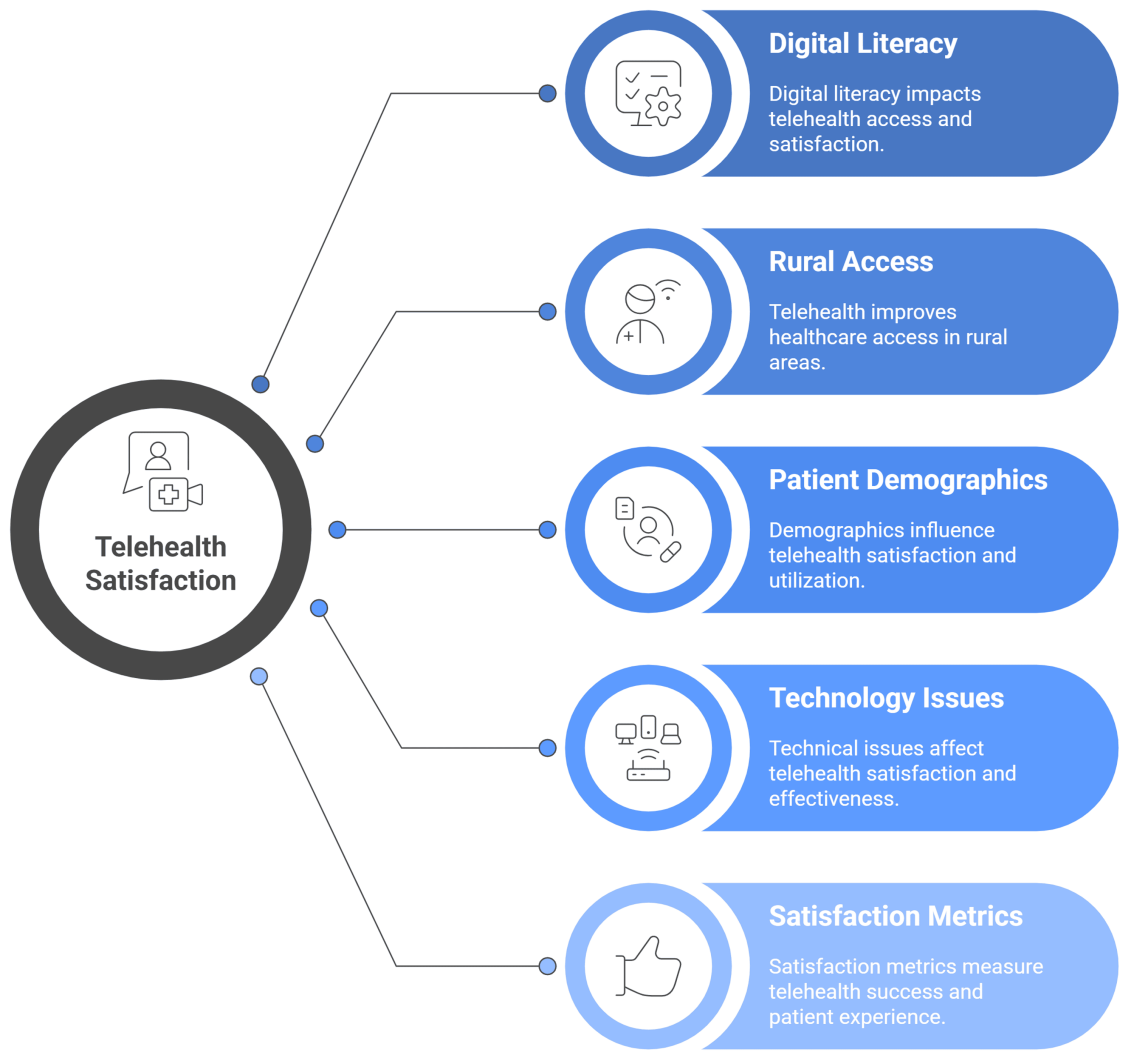
Community perspectives and satisfaction regarding . Image credit: Momen Abdelglil. Sources: [40-56].

Some obstacles to the use of telemedicine exist at the patient level, as observed in older patients, in particular, who may struggle to adopt the necessary technology and devices for telemedicine [54,55]. Lack of knowledge and access to these resources may cause healthcare workers to struggle with teleworking and telehealth. Managing virtual consultations, taking part in remote team meetings, staying disciplined, reducing distractions, overcoming feelings of loneliness, and setting up a formal home office are some of these difficulties (Figure 2) [56].

One obstacle to the adoption of telehealth is a lack of patient trust in virtual care compared to traditional in-person appointments, as supported by research that suggests that patients from minority racial groups often prefer emergency department visits to telemedicine or other remote options [57]. Some minority groups are especially affected by this lack of trust, and their preexisting mistrust of healthcare, even in conventional in-person settings, can be exacerbated during virtual consultations because of the already precarious patient-clinician connection [57,58]. Patient outcomes in telehealth are positively influenced by trust in clinicians [59].

The different language may be a great barrier for telehealth adoption that as highlighted in non-English-speaking communities. Poor audio translation during video calls often leads patients to opt for less effective phone consultations instead of telehealth visits [60]. Incorporating translators into telehealth tools can solve these difficulties and enhance the telephone experience [22,60].

Certain illnesses, including an undescended testis, are very difficult to diagnose via telemedicine, necessitating in-person examinations for future cases. Another difficulty, though one that is easier to resolve, is the requirement for radiographic imaging. In these situations, some families obtained the necessary studies and sent them to the clinical team electronically or via mail before the video consultation, which aided the review process. However, coordinating this can be time-consuming for everyone involved [53].

Including persistent software problems is another obstacle for widespread adoption, as well as a scarcity of video-compatible devices among patients, unreliable access to high-speed internet, and significant disparities in individual technological literacy [61]. Both users and service providers encountered various technical and connectivity issues, such as poor audio/video quality during visits and difficulties logging into the mobile application [62].

Telemedicine is suitable for chronic diseases that require long-term follow-up. Its use expands in urology; validated tools for this patient population will be beneficial for future research that will always seek higher performance with suitable cost. Patients receiving post-radical prostatectomy care and PSA surveillance have reported significantly higher satisfaction with telemedicine, indicating its continued use even after the COVID-19 pandemic [13]. Conversely, telemedicine is not suitable for conditions necessitating a physical exam or in-depth discussions. Patients with sexual health diagnoses might also hesitate to use telemedicine due to privacy worries [27].

In light of the COVID-19 pandemic, distant and wireless management has become more essential. This is a technology that will probably be used permanently. Now is the time for governments and nations to consider using these advances, which can enhance rural populations' access to healthcare. Establishing the demands of the program, determining the target patients, and assembling a driven, competent group are all necessary for successful implementation, revealing that organizational transformation is just as significant as the technology itself [63].

## Conclusions

Telemedicine in urology improves access where specialists are scarce, reduces patient and system costs through fewer in-person visits and travel, and maintains high satisfaction for follow-up care (e.g., virtual stone clinics). Yet adoption is uneven due to digital divides, workflow and imaging logistics, language needs, and patient trust - barriers that risk widening inequities if unaddressed.

Looking ahead, sustained adoption requires health-system investment in digital competency training for clinicians and patients, standardized hybrid pathways (clear indications for virtual vs in-person care), and rigorous evaluation of cost-effectiveness and equity. Emerging tools-AI-enabled triage and remote monitoring, integration with wearables, and tele-mentoring/robotics for selected procedures-should be developed and tested with attention to safety, privacy, and interoperability. Priorities include multilingual access, accessible device/internet options, and trust-building strategies in underserved communities.

As a narrative synthesis, findings may reflect selection and reporting bias; heterogeneity across study designs and outcomes limits comparability; and reliance on English-language sources may omit relevant evidence. These constraints underscore the need for prospective, diverse, and methodologically consistent studies to confirm generalizability and guide equitable implementation.
